# Supplementary material for: Quasi-periodic migration of single cells on short microlanes
Source: PLoS One. 2020 Apr 13;15(4):e0230679. doi: 10.1371/journal.pone.0230679 (PMC7153896; doi:10.1371/journal.pone.0230679)
Supplement: S1 Table — (DOCX) [file pone.0230679.s001.docx]

**Table S1. Parameters used for the computational simulation in this work.** The simulation conditions are used for all single cells.

| Name | Function | Value |
| --- | --- | --- |
| $\boldsymbol{R}$ | signaling radius | 4 |
| $\boldsymbol{\Delta\epsilon}$ | cell polarizability | 75 |
| $\boldsymbol{\kappa}_{\boldsymbol{P}}$ | cell contractility (perimeter stiffness) | 0.06 |
| $\boldsymbol{\mu}$ | cytoskeletal response rate | 0.02 |
| $\boldsymbol{\epsilon}_{\boldsymbol{0}}$ | average polarization field | 1500 |
| $\boldsymbol{\kappa}_{\boldsymbol{A}}$ | cell contractility (area stiffness) | 0.18 |
| $\boldsymbol{T}$ | effective temperature | 25 |
