# Supplementary material for: Quasi-periodic migration of single cells on short microlanes
Source: PLoS One. 2020 Apr 13;15(4):e0230679. doi: 10.1371/journal.pone.0230679 (PMC7153896; doi:10.1371/journal.pone.0230679)
Supplement: S2 Table — (DOCX) [file pone.0230679.s002.docx]

**Table S2. Number of analyzed cells for different lengths of microlane with round tips.**

| Length (µm) | L=70 | L=120 | L=170 | L=220 | L=270 |
| --- | --- | --- | --- | --- | --- |
| N_cell_ | 95 | 114 | 100 | 80 | 103 |
