# Supplementary material for: Quasi-periodic migration of single cells on short microlanes
Source: PLoS One. 2020 Apr 13;15(4):e0230679. doi: 10.1371/journal.pone.0230679 (PMC7153896; doi:10.1371/journal.pone.0230679)
Supplement: S3 Table — (DOCX) [file pone.0230679.s003.docx]

**Table S3. Number of analyzed cells for of microlanes with different geometric tips.**

| Microlanes | Round | Blunt | Concave | Sharp |
| --- | --- | --- | --- | --- |
| N_cell_ | 50 | 51 | 52 | 51 |
