# Supplementary material for: Quasi-periodic migration of single cells on short microlanes
Source: PLoS One. 2020 Apr 13;15(4):e0230679. doi: 10.1371/journal.pone.0230679 (PMC7153896; doi:10.1371/journal.pone.0230679)
Supplement: S1 Fig — (DOCX) [file pone.0230679.s004.docx]

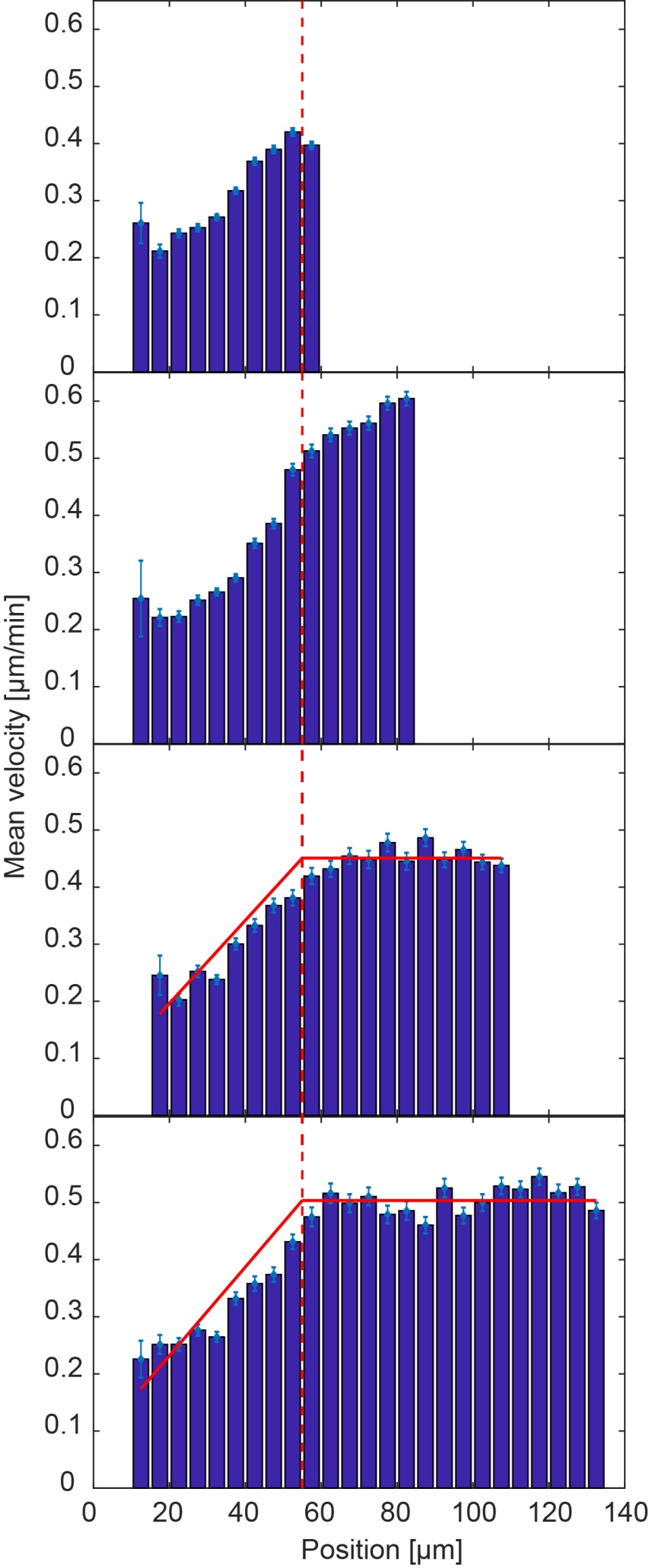


**Fig. S1 Mean cell velocity as a function of the distance to the nearest tip in microlanes of different lengths.** Top to bottom: 120, 170, 220, 270 µm. The velocity is averaged over the whole cell population in regions of width 5 µm. For 220 and 270 µm stripes, a clear plateau of constant velocity is visible towards the middle of the stripe. The transition from the plateau to the reversal area (dotted line) is found by a maximum likelihood “changepoint” analysis that finds changes between different linear regimes (red lines).
