# Supplementary material for: Quasi-periodic migration of single cells on short microlanes
Source: PLoS One. 2020 Apr 13;15(4):e0230679. doi: 10.1371/journal.pone.0230679 (PMC7153896; doi:10.1371/journal.pone.0230679)
Supplement: S2 Fig — (DOCX) [file pone.0230679.s005.docx]

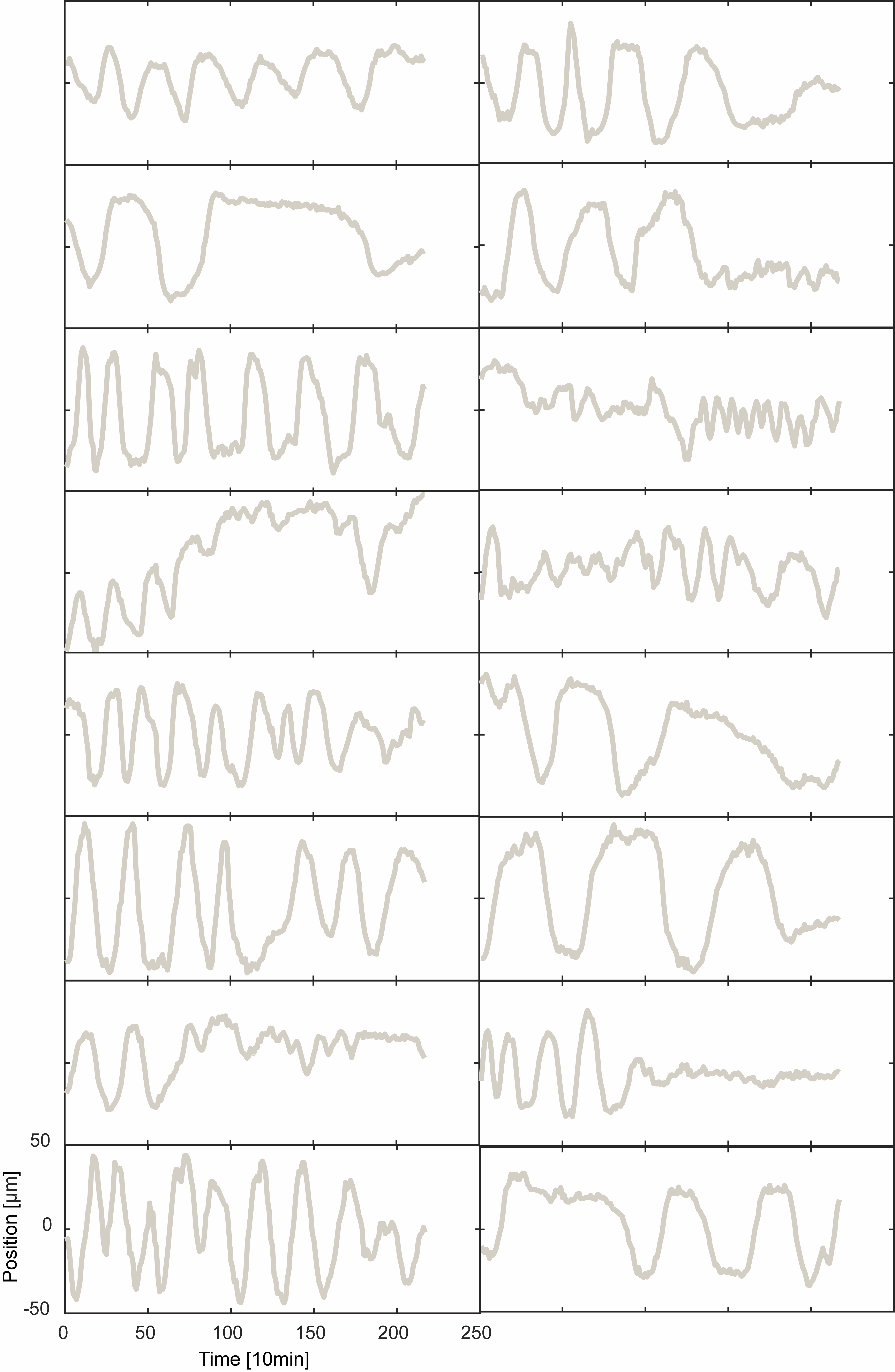


**Fig S2. Additional exemplary trajectories of cells with different frequencies on the microlane L=120** **µm.** Trajectory of cell nucleus tracked over the course of 36 h showing quasi-periodic alternations between directed migration and repolarization (width W = 20 µm, 10 min time intervals).
