# Supplementary material for: Quasi-periodic migration of single cells on short microlanes
Source: PLoS One. 2020 Apr 13;15(4):e0230679. doi: 10.1371/journal.pone.0230679 (PMC7153896; doi:10.1371/journal.pone.0230679)
Supplement: S1 File — (DOCX) [file pone.0230679.s013.docx]

## S1. Computer simulation

In our previous work, we have extended the original Cellular Potts model [1, 2] to better understand the mechanisms underlying cell locomotion. The simulations in this study are based on our previous work [3-5]. For convenience, we will recapitulate the essence of our model. For a more detailed and in-depth description of the model, we refer the reader to [4].

In our model, we consider the time evolution of the contact area between a cell and the two-dimensional surface that the cell migrates on. From now on, we will refer to this contact area simply as “cell”, which has area $A$ and perimeter $P$. We discretize each cell into a simply connected set of hexagons and associate its configuration with an energy, $H=H_{\text{cont}}+H_{\text{cyto}}$, which has contributions from cell contractility and from cell polarizability. Cell contractility is captured by the term

$$\mathcal{H}_{\text{cont}}=\kappa_{A}A^{2}+\kappa_{P}P^{2},$$

which assigns an energetical cost for increasing cell area or cell perimeter. Cell-substrate adhesions as well as forces exerted via actin polymerization and actomyosin contractility are captured by the term

$$\mathcal{H}_{\text{cyto}}=-\sum_{x} \epsilon\left( \boldsymbol{x},t \right),$$

where $\epsilon\left( \boldsymbol{x},t \right)$ is a spatially resolved scalar *polarization field*. We assume that the polarization field has both an upper bound (due to limited protein availability) and a lower bound (minimal adhesion energy): $\epsilon\left( \boldsymbol{x},t \right)\in\left[ \epsilon_{0}-\Delta\epsilon/2\ldots\epsilon_{0}+\Delta\epsilon/2 \right]$. Then, the time evolution of the cell is governed by a Metropolis algorithm with an effective temperature $T$ and gradually approaches the minimum of its associated energy $H$, by making new cell-substrate contacts (protrusion) or by detaching from the substrate (retraction).

We break detailed balance by introducing two prototypic feedback loops that serve to approximate the underlying chemical pattern formation:

1. Protrusions and the associated positive mechanochemical signaling reinforce the polarization field (cell adhesions and cell cytoskeleton) within a radius $R$. There, the polarization field exponentially approaches its upper bound, $\epsilon\to\epsilon_{0}+\Delta\epsilon$, with a rate $\mu$.
2. Retractions and the associated negative mechanochemical signaling weaken the polarization field (cell adhesions and cell cytoskeleton) within a radius $R$. There, the polarization field exponentially approaches its lower bound, $\epsilon\to\epsilon_{0}-\Delta\epsilon$, with a rate $\mu$.

In the absence of mechanochemical signals, the polarization field exponentially approaches its neutral state, $\epsilon\to\epsilon_{0}$, with a rate $\mu$.

Micropatterning of the substrate is achieved in the simulations by superimposing the polarization field with a second spatially varying scalar field $\varphi$, which reflects an offset in the cell-substrate binding energy. Here, we implement the micropattern by introducing no-go areas, $\varphi\to-\infty$ , which the cell cannot explore.

Our extended Cellular Potts model has several control parameters. The average polarization field, $\epsilon_{0}$, and the bulk stiffness, $\kappa_{A}$, jointly determine cell size. Cell dynamics is controlled by the ratio between the polarizability of the cell, $\Delta\epsilon$, and the membrane stiffness, $\kappa_{P}$, as well as the signaling range, $R$, and the cytoskeletal rate, $\mu$. The overall stochasticity of the system can be tuned by the effective temperature, $T$, which can also be absorbed by rescaling the membrane stiffness and the perimeter stiffness. In our previous work, we have investigated how cell behavior depends on these parameters[4]. In the current project, a finer cell discretization of the cell than that in[4] was necessary to resolve the micropattern tips. We achieved this by increasing the average polarization field compared to our previous work; note that it is equally valid to decrease the bulk stiffness[4]. Then, to account for the observed persistent cell motion and stochasticity in the experiments, we increased cell polarizability and the effective temperature. Furthermore, we reduced the signaling radius so that cells can form multiple competing lamellipodia. Lastly, we decreased the polarization update rate to account for a higher temporal resolution of the simulations.

# References

1. Graner F, Glazier JA. Simulation of biological cell sorting using a two-dimensional extended Potts model. Physical review letters. 1992;69(13):2013.

2. Glazier JA, Graner F. Simulation of the differential adhesion driven rearrangement of biological cells. Physical Review E. 1993;47(3):2128.

3. Segerer FJ, Thüroff F, Alberola AP, Frey E, Rädler JO. Emergence and persistence of collective cell migration on small circular micropatterns. Physical review letters. 2015;114(22):228102.

4. Thueroff F, Goychuk A, Reiter M, Frey E. Bridging the gap between single cell migration and collective dynamics. bioRxiv. 2019:548677.

5. Goychuk A, Brückner DB, Holle AW, Spatz JP, Broedersz CP, Frey E. Morphology and Motility of Cells on Soft Substrates. arXiv e-prints [Internet]. 2018 August 01, 2018. Available from: <https://ui.adsabs.harvard.edu/abs/2018arXiv180800314G>.
