# Supplementary material for: Quasi-periodic migration of single cells on short microlanes
Source: PLoS One. 2020 Apr 13;15(4):e0230679. doi: 10.1371/journal.pone.0230679 (PMC7153896; doi:10.1371/journal.pone.0230679)
Supplement: S2 File — (DOCX) [file pone.0230679.s014.docx]

## S2. Definition of reversal area

To study the cell repolarization at the edges separately from the free cell migration in the middle of the micropattern, we divided the stripe into two different regions. We distinguish a reversal area $A_{0}$of boundary $\xi_{i}$ at the tips of the stripe and a running area in the center.

We observed that the distance of the nucleus to the tip of the lane during repolarization varies substantially for different cells. Thus, it is not trivial to find the size of reversal area. On the one hand, it has to be large enough that the center of the nucleus is within the reversal area for all cells when touching the tip of the stipe. On the other hand, it has to be as small as possible to clearly differentiate running and repolarization of cells.

To find an appropriate boundary$\xi_{0}$ for the microlane with round tips, we looked at the mean velocity (along the long axis of the stripe) (Fig. 2), which shows a plateau in the middle and decreasing velocity towards the ends. In the area where the velocity decreases, the cells presumably interact with the end, thus we use this to define the reversal area. To make the transitions easier to detect, we calculate the mean velocity in 5µm wide areas as a function of the distance to the ends (combined left and right side) (Fig. S1). For 220 and 270 µm stripes, the transition is clearly visible. We use the “findchangepts” function in Matlab, which uses a maximum likelihood approach to find the most probable “changepoint” between two linear regimes. The resulting value is $\xi_{0}$= 55 µm for the round geometry, leading to a reversal area $A_{0}$ = 1057 µm² for all geometries.

# References

1. Graner F, Glazier JA. Simulation of biological cell sorting using a two-dimensional extended Potts model. Physical review letters. 1992;69(13):2013.

2. Glazier JA, Graner F. Simulation of the differential adhesion driven rearrangement of biological cells. Physical Review E. 1993;47(3):2128.

3. Segerer FJ, Thüroff F, Alberola AP, Frey E, Rädler JO. Emergence and persistence of collective cell migration on small circular micropatterns. Physical review letters. 2015;114(22):228102.

4. Thueroff F, Goychuk A, Reiter M, Frey E. Bridging the gap between single cell migration and collective dynamics. bioRxiv. 2019:548677.

5. Goychuk A, Brückner DB, Holle AW, Spatz JP, Broedersz CP, Frey E. Morphology and Motility of Cells on Soft Substrates. arXiv e-prints [Internet]. 2018 August 01, 2018. Available from: <https://ui.adsabs.harvard.edu/abs/2018arXiv180800314G>.
